# A hybrid Harris Hawks Optimization with Support Vector Regression for air quality forecasting

**DOI:** 10.1038/s41598-025-86275-6

**Published:** 2025-01-17

**Authors:** Essam H. Houssein, Meran Mohamed, Eman M. G. Younis, Waleed M. Mohamed

**Affiliations:** Faculty of Computers and Information, Minia University, Minia, Egypt

**Keywords:** Support Vector Regression (SVR), Particulate Matter, Air Quality, Metaheuristics, Harris Hawks Optimization (HHO), Climate sciences, Computational science

## Abstract

This paper proposes a hybridized model for air quality forecasting that combines the Support Vector Regression (SVR) method with Harris Hawks Optimization (HHO) called (HHO-SVR). The proposed HHO-SVR model utilizes five datasets from the environmental protection agency’s Downscaler Model (DS) to predict Particulate Matter ($PM_{2.5}$) levels. In order to assess the efficacy of the suggested HHO-SVR forecasting model, we employ metrics such as Mean Absolute Percentage Error (MAPE), Average, Standard Deviation (SD), Best Fit, Worst Fit, and CPU time. Additionally, we contrast our methodology with recently created models that have been published in the literature, such as the Grey Wolf Optimizer (GWO), Salp Swarm Algorithm (SSA), Henry Gas Solubility Optimization (HGSO), Barnacles Mating Optimizer (BMO), Whale Optimization Algorithm (WOA), and Manta Ray Foraging Optimization (MRFO). In particular, the proposed HHO-SVR model outperforms other approaches, establishing it as the optimal model based on its superior results.

## Introduction

Over the past twenty years, meta-heuristic optimization approaches have gained immense popularity. Most of them, such as SSA^1^, EO^2^, HHO^3^, GWO^4^, BMO^5^, MRFO^6^, WOA^7^, AO^8^, AOA^9^, and HGSO^10^, are well acknowledged by scientists of multiple disciplines in addition to machine learning experts. These optimization approaches have been applied to a wide range of study topics and have been used to solve numerous optimization issues, including jobs that are non-linear, non-differentiable, or computationally demanding with many local minima. In addition, a substantial number of scientific papers have been conducted on these methodologies. The surprising popularity of metaheuristics can be attributed to four main factors: clarity, pliability, derivation-free processes, and the ability to avoid local optima^4,11^. Typically, these techniques can be categorized into four distinct groups^12^: evolution-based, physics-inspired, swarm-based^13,14^, and human-based algorithms.

Evolution-based models: integrate mechanisms such as chemical sensing and movement, reproductive processes, removal, distribution, and movement patterns.^15^. Among these, the Genetic Algorithm (GA) stands out as a common and powerful evolutionary technique^16^. In particular, GA does not require derivatives, unlike mathematical optimization methods. By emulating successful strategies, GA improves populations through efficient tactics such as escaping local optima. Over time, different approaches were suggested to improve the efficiency of GA. Furthermore, other evolutionary techniques have emerged based on the success of GA^17^, including Evolutionary Programming (EP)^18^, Differential Evolution (DE)^19^, Evolutionary Strategies (ES)^20^, and the Artificial Algae Algorithm (AAA)^21^.

Physics-based: simulate the physical rules governing our planet. One of the most recognized algorithms in this category is Simulated Annealing (SA)^22^. SA approximates physical material thermodynamics. The process of annealing, which involves cooling and crystallizing hot metals, is used to reduce electricity consumption. Additionally, several new physics-inspired algorithms, such as the Gravitational Search Algorithm (GSA)^23^. Lévy Flight Distribution (LFD)^24^, Archimedes optimization algorithm (AOA)^25^, have been established.

Swarm-based algorithms: strive to replicate the social tendencies observed in creatures, such as self-organizing mechanisms and the assignment of work duties.^26^. Two notable case studies in this domain are Particle Swarm Optimization (PSO)^27^ and Ant Colony Optimization (ACO)^28^. PSO, inspired by bird flocking activities, adjusts every agent based on both its best individual performance and the best global within the group. ACO, on the other hand, draws inspiration from ant swarms’ foraging habits and the diminishing strength of pheromones over time. Ants use this approach to find the most efficient path from their nest to a food source. In addition, other swarm-inspired techniques include Glowworm Swarm Optimization (GSO)^29^, Harris Hawks Optimization (HHO) , and cuckoo search (CS)^30^, as well as Artificial Ecosystem-based Optimization (AEO)^31^.

Human-based algorithms: mainly derived from human behavior where each individual has a unique way of accomplishing tasks that can impact their overall performance. Which becomes a motivation for researchers to improve the models^32^. The most well-known human-based algorithm is called Teaching-Learning-Based Optimization (TLBO), and it was developed to simulate the classroom interactions between the instructor and his students^33^. Human Mental Search (HMS)^34^ was designed by simulating human behavior versus online auction platforms. Doctor and Patient optimization algorithm (DPO)^35^ was designed with consideration for interactions between healthcare providers and patients, including illness prevention, examination, and therapy.

The No Free Lunch Theorem^36^ in optimization states that no single optimizer performs optimally across all optimization scenarios. Consequently, the pursuit of robust swarm-inspired optimizers has become a driving force for researchers aiming to tackle intricate real-world problems^37,38^ . In this study, we propose eight hybrid frameworks that incorporate modern metaheuristic techniques. These frameworks are specifically designed to fine-tune support vector regression parameters for forecasting the daily maximum concentration of Particulate Matter ($PM_{2.5}$).

According to the literature, various methods have been employed with different characterizations. Among these, optimization methods have proven their efficiency in solving $PM_{2.5}$ forecasting problems compared to traditional approaches. However, SVR and optimization methods have been underutilized, despite their potential to provide more reliable solutions for forecasting. Existing search methods often face limitations in performance, model complexity, and time required to build and solve the problem. Consequently, realizing accurate calculation results can be challenging. Furthermore, as highlighted in^39^, a significant gap in this problem lies in the complex process of model establishment, which necessitates a comprehensive understanding of each variable’s impact on the target value. Unfortunately, some factors may be overlooked during implementation^40^. Although most current studies focus on non-linear models for $PM_{2.5}$ forecasting, only a few have explored advanced machine learning and optimization techniques. This claim is reinforced by^41^, in which a cutting-edge optimization technique is utilized as a prediction system relying on unstructured data, leading to more accurate and coherent forecasts. In general, the consensus in the literature is that the $PM_{2.5}$ forecasting problem is highly intricate and requires an efficient approach^42^.

This study compares the proposed HHO hybrid model with other recognized metaheuristic optimization techniques. These encompass GWO, WOA, SSA, BMO, HGSO, MRFO, and EO. Table 1 shows a summary of each of the algorithms, detailing their core principles, strengths, and limitations.

**Table 1:** Summary of comparative algorithms used in this study.

Algorithm	Principle	Strengths and Limitations
HHO	Inspired by the predatory behavior of hawks using Levy flight and soft-hard besiege strategies	Strong exploration capabilities but requires careful parameter tuning to balance search efficiency
GWO	Mimics the social hierarchy and hunting strategies of grey wolves	Simple to implement, with effective convergence, but prone to stagnation in complex search spaces
WOA	Based on the bubble-net hunting behavior of humpback whales	Excels in exploration, but lacks diversity in the exploitation phase, reducing solution refinement
SSA	Models the swarming behavior of salps in the ocean	Efficient for smaller-scale problems, but convergence becomes slower for high-dimensional or complex functions
BMO	Simulates the mating behavior of barnacles	High diversity and global search ability but computationally expensive, particularly for large-scale problems
HGSO	Derives from Henry’s law and gas solubility in liquids	Demonstrates strong convergence properties, but performance may depend on initial population quality
MRFO	Inspired by the foraging strategies of manta rays	Effectively balances exploration and exploitation, making it robust across various optimization problems
EO	Physics-based model utilizing dynamic mass balance and equilibrium states	Combines robust exploration and exploitation, highly competitive in benchmark functions, but sensitive to parameter tuning and slightly less effective in composite functions

The paper presents the following contributions:

- The study introduces a hybrid model that employs Support Vector Regression (SVR) with Harris Hawks Optimization (HHO) for the accurate prediction of $PM_{2.5}$ concentrations.
- The effectiveness of the suggested approach is evaluated by the Mean Absolute Percentage Error (MAPE), Average, Standard Deviation (SD), Best Fit, Worst Fit, and CPU time.
- All models were trained with new real data from the Centers for Disease Control and Prevention’s National Environmental Public Health Tracking Network (1001, 1003, 1005, 1007, 1009) which is a governmental organization.

The paper is organized as follows: “Materials and methods” section provides an in-depth discussion of the general principles underlying the SVR model, along with an exploration of the HHO algorithm. In “The proposed HHO-SVR model” section, we delve into the proposed HHO-SVR model, detailing its configuration and settings. Moving on to “Experimental results analysis and discussion” section, we present the definition, analysis, and measurement criteria employed to assess precision, as well as an interpretation and thorough discussion of the obtained outcomes. Finally, “Conclusion and future directions” section offers the conclusion, summarizing the key findings and implications (Table 2).

**Table 2:** Sample of the studies related to $PM_{2.5}$ forecasting.

Study	Publishing year	Dataset	Period	Goal	Evaluation metric	Method
^43^	2019	the Greater London Area Pollution Data	2016:2017	$O_3$, $PM_{10}$	MAE, RMSE, AI, $R^2$	CEeSNN
^44^	2019	Collected by auther	16 mos	$PM_{2.5}$	MAE, $R^2$	Kernel based linear regression, Bayesian based
^45^	2020	Beijing, Wuhan, Shanghai, Guangzhou, Chengdu	2014	$PM_{2.5}$	$R^ 2$	MLR, GWR, RF, GRNN
^46^	2020	China	2014	$PM_{2.5}$	$R^2$, RMSE, MPE, RPE	GTWNN
^47^	2020	Jinan, Nanjing, Chongqing	2017:2018	$PM_{2.5}$,$PM_{10}$, $SO_2$,AQI	MAE, RMSE, MAPE, IA, U1,U2, *R*	ICEEMDAN-MOHHO-ELM
^48^	2020	Central Pollution Control in India		$PM_{2.5}$	$R^2$, MAE, MAPE, RMSE	WANFIS-PSO
^49^	2020	CPCB	2016:2018	$PM_{2.5}$	MSE, Pearson Correlation	ANN, SVM
^50^	2020	Beijing, Guangzhou, Shanghai	2016	$PM_{2.5}$, $PM_{10}$	MAE, RMSE, MAPE, TIC, VAR,IA, DA	FCFS
^51^	2020	Beijing	2010:2014	$PM_{2.5}$	RMSE	AE-BiLSTM
^52^	2020	TAQMN	2012: 2017	$PM_{2.5}$	RMSE, MSE, MAE, $R^2$	Gradient Booting Regression
^53^	2021	China (14 cities)	2014:2018	$PM_{2.5}$	RMSE, MAE	FDN-Learning
^54^	2021	Beijing	2015:2017	$PM_{2.5}$	MAPE, MAE, RMSE	CEEMDAN-DeepTCN
^39^	2021	Beijing, Shanghai, Wuhan, Guangzhou	2019	$PM_{2.5}$	MAE, MAPE, RMSE	GB-ELM-MIMO-ECM, MAdaBoost-ELM-MIMO-ECM, GB-ELM-Recursive-ECM, MAdaBoost-ELM-Recursive-ECM
^55^	2021	BJ2014	2014:2015	$PM_{2.5}$	MAE, RMSE, SMAPE	SpAttRNN
		BJ2017	2017:2018			
		Meteorological , POI				
^56^	2021	Beijing	2014:2015	$PM_{2.5}$	RMSE, MAE, $R^2$	ST-CausalConvNet
^41^	2022	Bei-Shang-Guang-Shen	2020	$PM_{2.5}$	MAPE, MAE, RMSE, DA	mRMR, BPNN, ELM, GRNN, BiLSTM, MOWCA
^57^	2022	Collected by auther		$PM_{2.5}$, CO, $SO_2$, $O_3$, $H_{2}S$, $NO_2$,$PM_{10}$	MSE	LSTM
^58^	2022	Beijing, Tianjin, Shanghai, Chongqing	2018:2019	$PM_{2.5}$, $PM_{10}$	MAPE, MAE, RMSE, NRMSE, $R^2$	RLMD-ARIMA- RVMcom-MW
^59^	2022	Japanese ministry of environment.	2014	$PM_{2.5}$, $NO_2$	RMSE, ME, MAE, $R^2$	LightGBM, RF,XGBoost
^60^	2022	China’s National Ambient Air Quality Monitoring Network	2015:2020	$PM_{2.5}$	$R^2$, MAE, MAPE, RMSE	RF
^61^	2022	CNEMC	2013:2020	$PM_{2.5}$	RMSE, $R^2$, MAE, PRE	STENN
^62^	2022	Utrecht, NL	2020	$PM_{2.5}$	RMSE, MAE, IA, *R*, Accuracy, NMB, NMSD	AVGAE, GRF
		Antwerp, BE	2021	$NO_2$		
		Oakland, US	2019	$NO_2$		
^40^	2023	Beijing $PM_{2.5}$	2010:2014	$PM_{2.5}$	RMSE, MAE	CRINet
		Beijing Shunyi-Station	2013:2017	$SO_2$, $NO_2$, $PM_{2.5}$, $PM_{10}$, $O_3$		
^63^	2023	Lanzhou	2020:2022	$PM_{2.5}$	MAPE, RMSE, MAE, SMAPe, Dstat	VMD-ARIMA-CNN-TCN
^64^	2023	India	2015:2020	$PM_{2.5}$, $PM_{10}$, $NO_2$, $SO_2$, $O_3$	MAPE, $R^2$	SVR, XGBOOST
^65^	2023	University of California Irvine (UCI) Beijing, China	2010:2014	$PM_{2.5}$	RMSE, $R^2$, MAE, PRE	RBOSR
^42^	2023	Xi’an	2018:2020	$PM_{2.5}$	RSE, CORR, RMSE, MAE	STF-Net
		Beijing	2013:2017			

## Materials and methods

In the following section, the fundamental concepts of SVR along with HHO are addressed.

### Support Vector Regression (SVR)

SVR is a data-driven approach that originated from Support Vector Machine (SVM). SVR is used for regression tasks by implementing the $\varepsilon$–insensitive loss function. For further information and a detailed description of support Vector Machine (SVM), you can refer to^66^. Suppose that the learning variables are assigned to $D=\left\{ {\left( {{x_i},{y_i}}\right) }\right\}$, where the input is ${x_i}\in R$, and the output is ${y_i} \in R$ for $i=1,2,3, \cdots , N$, and the sample number is *N*. The goal of SVR is to determine a functional relationship, denoted as *f*(*x*), that links the input variables $x_{i}$ to the output variable $y_{i}$. This is done without any prior knowledge of the joint distribution *P* of the variables (*x*, *y*). The formula in the linear case is $f(x)= \langle {w, x}\rangle + b$, where *w* is the weight and *b* is the constant coefficient respectively. A non-linear mapping, denoted as ($\Phi$), is utilized to convert a hard non-linear task into a more feasible linear task. Regression function is presented in Eq. (1).

1 $$\begin{aligned} f(x) = \left\langle {w,x} \right\rangle + b \end{aligned}$$

The *f*(*x*) tend to adjust the learning sets. with flexibility and aim for a minimal slope by reducing the standard value of *w* to avoid overfitting issues. In order to address constraints that would otherwise be impossible to overcome, introducing two slack variables, denoted as $\xi$ and ${\xi }_{i}^{*}$. The feasibility of convex optimization in this context relies on the existence of a function that accurately adjust all pairs of data $({x_i},{y_i})$ with a suitable accuracy level, denoted as $\varepsilon$. The problem should be designed as a convex optimization job, as depicted in Eq. (2).

2 $$\begin{aligned} \begin{array}{l} {\textrm{minimize}}\,\,\frac{1}{2}{\left\| w \right\| ^2} + c\sum \limits _{i = 1}^l {\left( {{{\xi }_i} + {\xi }_{i}^{*} } \right) } \\ {\textrm{subject to}}\,\,\left\{ {\begin{array}{*{20}{c}} {\left\langle {w,\Phi \left( x \right) } \right\rangle + b - y \le \varepsilon + {{\xi }_i}}\\ {{y_i} - \left\langle {w,\Phi \left( x \right) } \right\rangle - b \le \varepsilon + {\xi }_i{*} }\\ {{{\xi }_i},{\xi }_{i}^{*} \le 0} \end{array}} \right. \end{array} \end{aligned}$$

where *C* is the penalty factor constant, and ${{\xi _i},{\xi }_{i}^{*}}$ Denotes the difference between the anticipated and the intended values.

More easily, the matter of optimization in its dual forms can be solved. Using $K\left( {{x_i},{x_j}} \right) = {\Phi ^T}\left( {{x_i}} \right) \Phi \left( {{x_j}} \right)$ as a direct substitute for the saddle point constraint instead of $\Phi (.)$ explicitly, Eq.(3) yields the kernel version of the dual optimization problem by removing the dual variables ${{\eta }_i},\eta _{i}^{*}$. The kernel function that satisfies the mercer condition is denoted as $K\left( {x,x'} \right)$^67^.

3 $$\begin{aligned} \begin{array}{l} {\textrm{minimize}}\,\,\frac{1}{2}\sum \limits _{i,j = 1}^l {\left( {\alpha _{i}^{*} - {\alpha _i}} \right) \left( {\alpha _{j}^{*} - {\alpha _j}} \right) } K\left( {{x_i} \cdot {x_j}} \right) \\ + \varepsilon \sum \limits _{i = 1}^l {\left( {\alpha _{i}^{*} + {\alpha _i}} \right) - \sum \limits _{i = 1}^l {y\left( {\alpha _{i}^{*} - {\alpha _i}} \right) } } \\ {\textrm{subject to}}\,\,\left\{ {\begin{array}{*{20}{c}} {\sum \limits _{i = 1}^l {\left( {\alpha _{i}^{*} - {\alpha _i}} \right) = 0,} }\\ {0 \le {\alpha _i},\alpha _{i}^{*} \le C} \end{array}} \right. \,\, \end{array} \end{aligned}$$

Due to the fact that the Lagrange multipliers are $\alpha _i$ and $\alpha _{i}^{*}$, *w* is directly solved in Eq. (4) after resolving the two original optimization problems. Support Vectors (SVs) are denoted by the positive and non-zero samples $\alpha _i$ and $\alpha _{i}^{*}$. At the optimal solution, the product of two variables and the constraints should vanish, achieved through the use of Karush Kuhn Tucker (KKT) restrictions. These restrictions define the necessary and satisfactory conditions for achieving a global optimum. We determine the parameter *b* in Eq. (5). The function *f*(*x*) is modified in the support vector expansion shown in Eq. (6). A function complexity depends solely on the number of SVs and is independent of the size of the input space.

4 $$\begin{aligned} w= & \sum \limits _{i = 1}^l {\left( {\alpha _{i}^{*} - {\alpha _i}} \right) {x_i}} \end{aligned}$$

5 $$\begin{aligned} b= & {y_j} - \left\langle {w,\Phi \left( x \right) } \right\rangle - \varepsilon \,for\,\,\,0 \le {\alpha _i} \le C\nonumber \\ b= & {y_j} - \left\langle {w,\Phi \left( x \right) } \right\rangle - \varepsilon \,for\,\,\,0 \le \alpha _{i}^{*} \le C \end{aligned}$$

6 $$\begin{aligned} f\left( x \right)= & \sum \limits _{i = 1}^l {\left( {\alpha _{i}^{*} - {\alpha _i}} \right) } K\left( {{x_i},x} \right) + b \end{aligned}$$

The SVR technique presents an intriguing topic on the seemingly random process of selecting a kernel for specific data patterns^68,69^. The gaussian RBF kernel is an implementation that works better than other kernel functions in terms of simplicity of use and effective mapping functionality. Therefore, in this article, $K\left( {x,x'} \right) = \exp \left( { - \frac{{{{\left\| {x - x'} \right\| }^2}}}{{2{\sigma ^2}}}} \right)$ represents the gaussian function of the RBF kernel. Two parameters are involved in the SVR method.The trade-off between the complexity of the function and the frequency at which an error is allowed is controlled by the *C* parameter. The $\sigma$ parameter controls the complexity of the model and shows the mapping of the translated input variables into feature space. As mentioned in^70^, it is consequently important to determine appropriate parameters and to choose the value of the $\sigma$ parameter more carefully than *C*. SVR pseudo-code is displayed by Algorithm 1.

**Algorithm 1:**
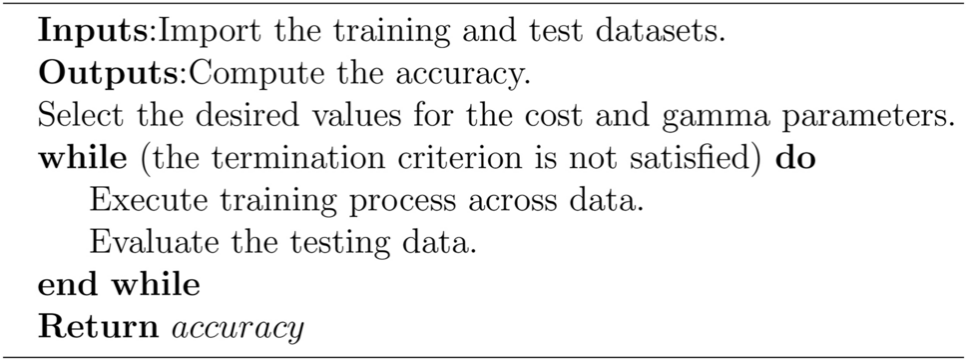
The pseudo-code of SVR model.

### Harris Hawks Optimization (HHO)

**Fig. 1:**
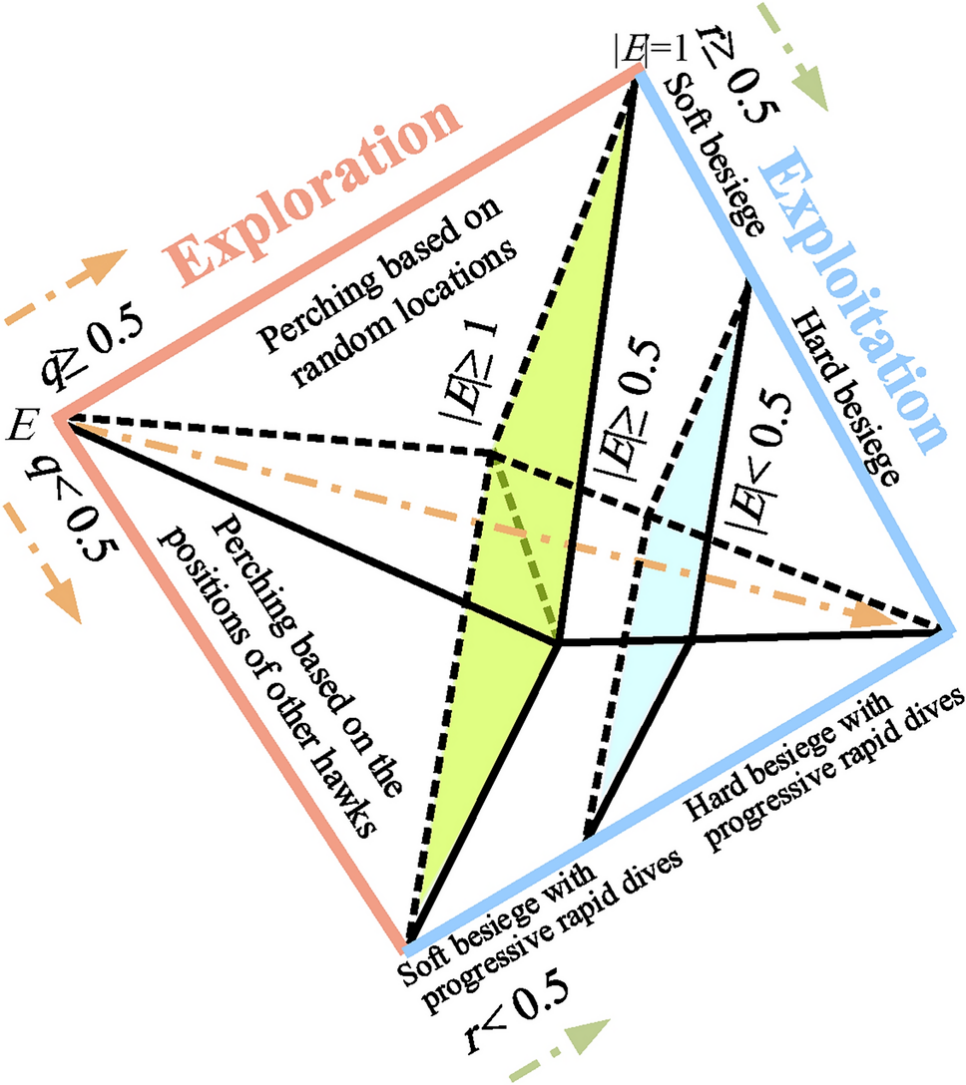
Different phases of HHO^3^.

Figure 1 shows all phases of HHO, which are described in the next subsections.

#### Exploration phase

In HHO, The Harris’ hawks exhibit a random perching behavior at various areas, employing two distinct strategies to detect and capture their prey.

7 $$\begin{aligned} X(t+1) = \left\{ \begin{matrix} X_{rand}(t)-r_{1}\left| X_{rand}(t)-2r_{2}X(t) \right| & q\ge 0.5 \\ (X_{rabbit}(t)-X_{m}(t))-r_{3}(LB+r_{4}(UB-LB)) & q<0.5 \end{matrix}\right. \end{aligned}$$

where $X(t+1)$ represent the position vector of the hawks in the next iteration *t*, $X_{rabbit}(t)$ denote the position of the rabbit, and *X*(*t*) is the current position vector of hawks. Additionally, we have random numbers $r_{1}$, $r_{2}$, $r_{3}$, $r_{4}$, and *q* q, which are uniformly distributed between 0 and 1 and are updated in each iteration. The variables *LB* and *UB* represent the lower and upper bounds for the hawk positions. Furthermore, $X_{rand}(t)$ corresponds to a randomly selected hawk from the current population, and $X_{m}$ represents the average position of the current hawks population. The average position of hawks is attained using Eq. (8):

8 $$\begin{aligned} X_{m}(t)=\frac{1}{N}\sum _{i=1}^{N}X_{i}(t) \end{aligned}$$

where $X_{i}(t)$ represents the position of each hawk at iteration *t*, whereas *N* represents the total number of hawks.

#### Exploration to exploitation transition

**Fig. 2:**
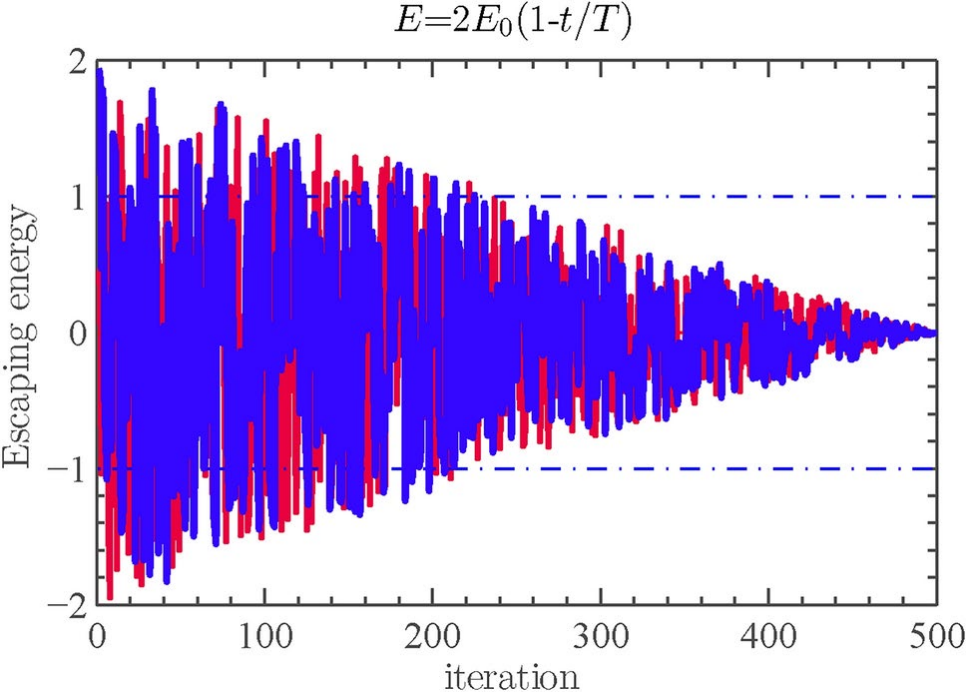
An illustration of the variable *E* during the execution of two runs and 500 rounds^3^.

To model this step, the energy of a rabbit is modeled as:

9 $$\begin{aligned} E=2E_{0}\left( 1-\frac{t}{T}\right) \end{aligned}$$

where *E* represents the amount of energy required for the prey to escape. The variable *T* represents the upper limit for the number of iterations, while $E_{0}$ represents the initial energy state of the prey. The temporal dynamics of *E* are also illustrated in Fig. 2.

#### Exploitation phase

In this phase, Harris’ hawks execute a surprise pounce on the prey identified in the preceding stage. Prey typically attempts to evade capture, resulting in diverse chasing behaviors in real-world scenarios. To model the attack phase, HHO incorporates four potential strategies based on the prey’s escape behaviors and the hawks’ pursuit tactics. The prey consistently strives to escape from threats, with the probability $r$ representing the likelihood of successful evasion ($r < 0.5$) or failure ($r \ge 0.5$) before the pounce. Regardless of the prey’s actions, the hawks employ either a hard or soft besiege to capture it, encircling the prey from multiple directions based on its remaining energy. In natural settings, the hawks progressively close in on the prey, enhancing their cooperative hunting success via a surprise pounce. Over time, the escaping prey becomes increasingly fatigued, allowing the hawks to intensify their besiege and capture the exhausted prey with greater ease. To simulate this strategy within the HHO algorithm, the $E$ parameter is utilized: a soft besiege occurs when $|E| \ge 0.5$, while a hard besiege is employed when $|E| < 0.5$.

*Soft besiege* The following rules are used to model this behavior:

10 $$\begin{aligned} X(t+1)= & \Delta X(t)-E\left| JX_{rabbit}(t)-X(t)\right| \end{aligned}$$

11 $$\begin{aligned} \Delta X(t)= & X_{rabbit}(t)-X(t) \end{aligned}$$

where $\Delta X(t)$ represents the difference between the position vector of the rabbit and its current location at iteration *t*, while $r_{5}$ is a random number between 0 and 1. The variable *J* is defined as $2(1-r_{5})$ and represents the random jump strength of the rabbit during the fleeing method, and its value undergoes random changes in each iteration to mimic the unpredictable nature of rabbit movements.

*Hard besiege* In this situation, the current positions are updated using Eq. (12):

12 $$\begin{aligned} X(t+1)=X_{rabbit}(t)-E \left| \Delta X(t) \right| \end{aligned}$$

**Fig. 3:**
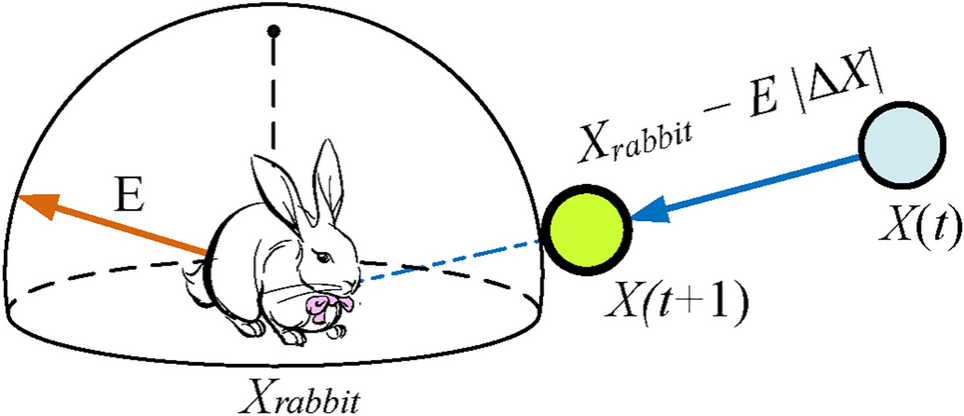
An illustration of all vectors in the context of hard besiege^3^.

A simple illustration of this step with one hawk is depicted in Fig. 3.

*Soft besiege with progressive rapid dives* We assume that in order to execute a soft besiege, the hawks can assess (decide) their next step in accordance with the following rule in Eq. (13):

13 $$\begin{aligned} Y=X_{rabbit}(t)-E\left| JX_{rabbit}(t)-X(t)\right| \end{aligned}$$

We assumed that they would use the following rule to dive based on the LF-based patterns:

14 $$\begin{aligned} Z=Y+S\times LF(D) \end{aligned}$$

where LF is the levy flight function, which is determined by applying Eq. (15), and *D* represents the problem’s dimension, while *S* is a random vector of size $1\times D$.

15 $$\begin{aligned} LF(x)=0.01\times \frac{u\times \sigma }{\left| v \right| ^{\frac{1}{\beta }}}, \sigma =\left( \frac{\Gamma (1+\beta )\times sin(\frac{\pi \beta }{2})}{\Gamma (\frac{1+\beta }{2})\times \beta \times 2^{(\frac{\beta -1}{2})})} \right) ^{\frac{1}{\beta }} \end{aligned}$$

where *u* and *v* represent random values between 0 and 1, and $\beta$ denotes a default constant set to 1.5.

Therefore, Eq. (16) can be used as the final strategy for updating the positions of hawks during the soft besiege phase.

16 $$\begin{aligned} X(t+1)=\left\{ \begin{matrix} Y & if F(Y)<F(X(t)) \\ Z & if F(Z)<F(X(t)) \\ \end{matrix}\right. \end{aligned}$$

where *Y* and *Z* are obtained using Eqs. (13) and (14).

**Fig. 4:**
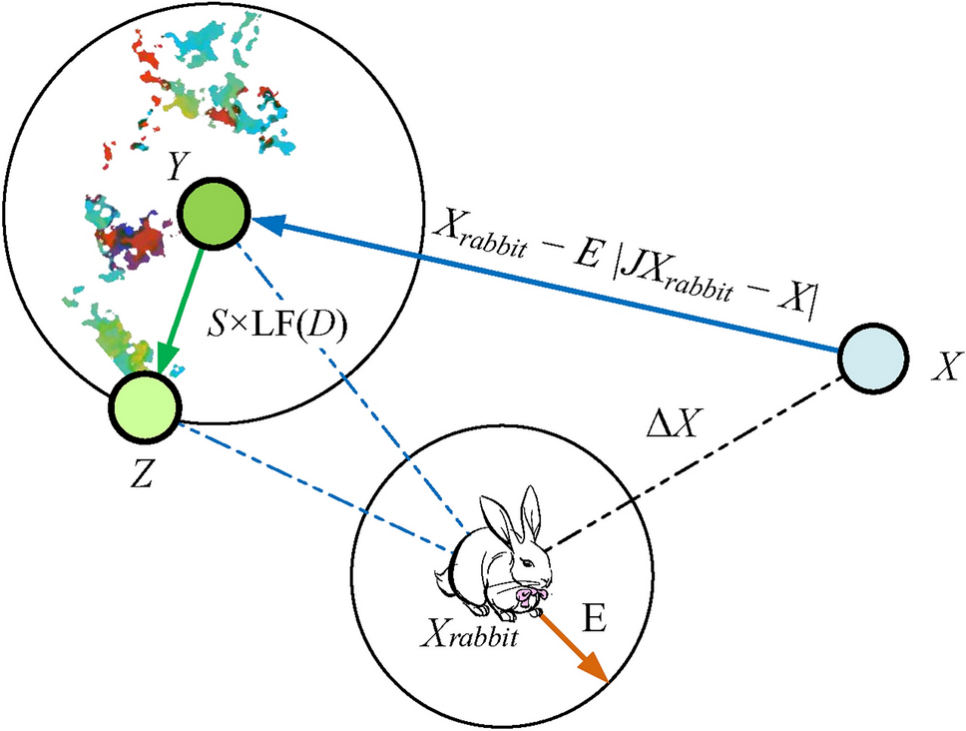
A illustration of all vectors in the context of soft besiege with progressive rapid dives^3^.

A simple illustration of this step for one hawk is demonstrated in Fig. 4.

*Hard besiege with progressive rapid dives* The following rule is performed in hard besiege condition:

17 $$\begin{aligned} X(t+1)=\left\{ \begin{matrix} Y & if F(Y)<F(X(t)) \\ Z & if F(Z)<F(X(t)) \\ \end{matrix}\right. \end{aligned}$$

where *Y* and *Z* are obtained using new rules in Eqs.(18) and (19).

18 $$\begin{aligned} Y= & X_{rabbit}(t)-E\left| JX_{rabbit}(t)-X_{m}(t)\right| \end{aligned}$$

19 $$\begin{aligned} Z= & Y+S\times LF(D) \end{aligned}$$

where $X_{m}(t)$ is obtained using Eq. (8).

**Fig. 5:**
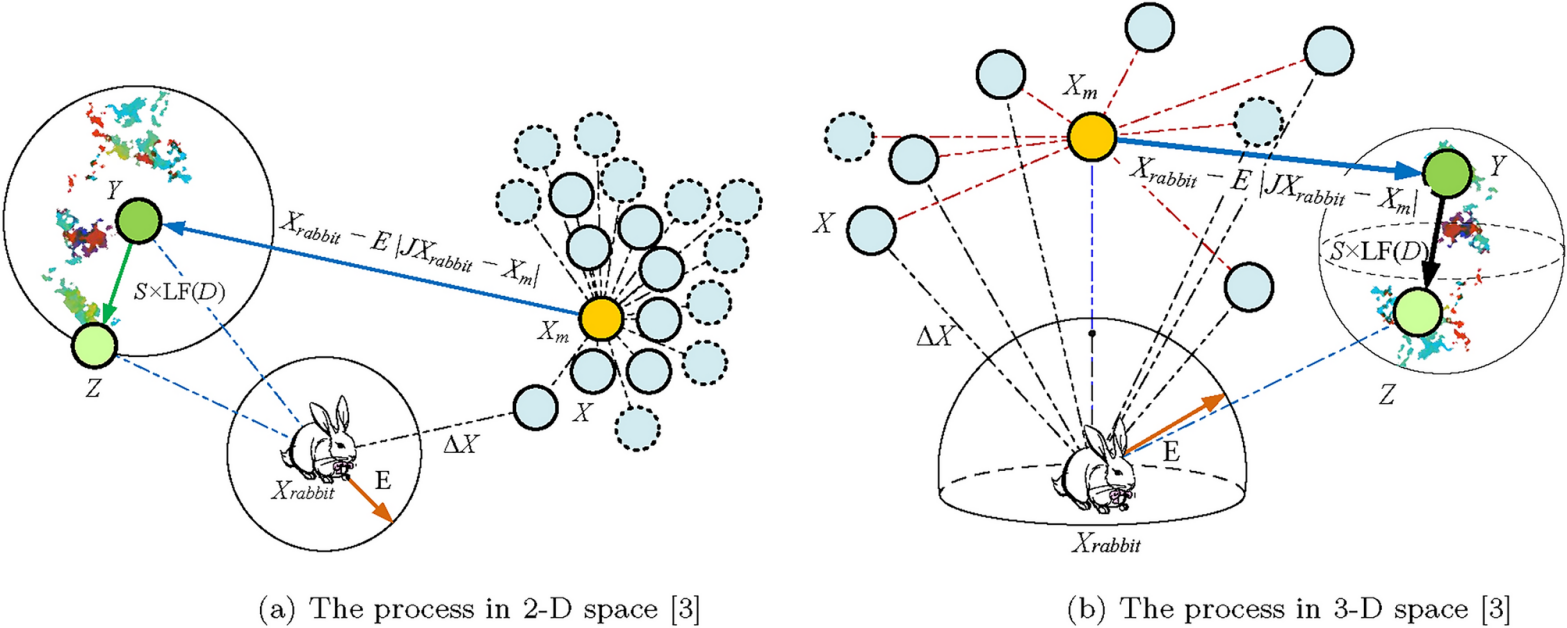
An illustration of all vectors in the context of hard besiege with progressive rapid dives in 2-D and 3-D spaces.

A simple illustration of this step is demonstrated in Fig. 5.

## The proposed HHO-SVR model

**Fig. 6:**
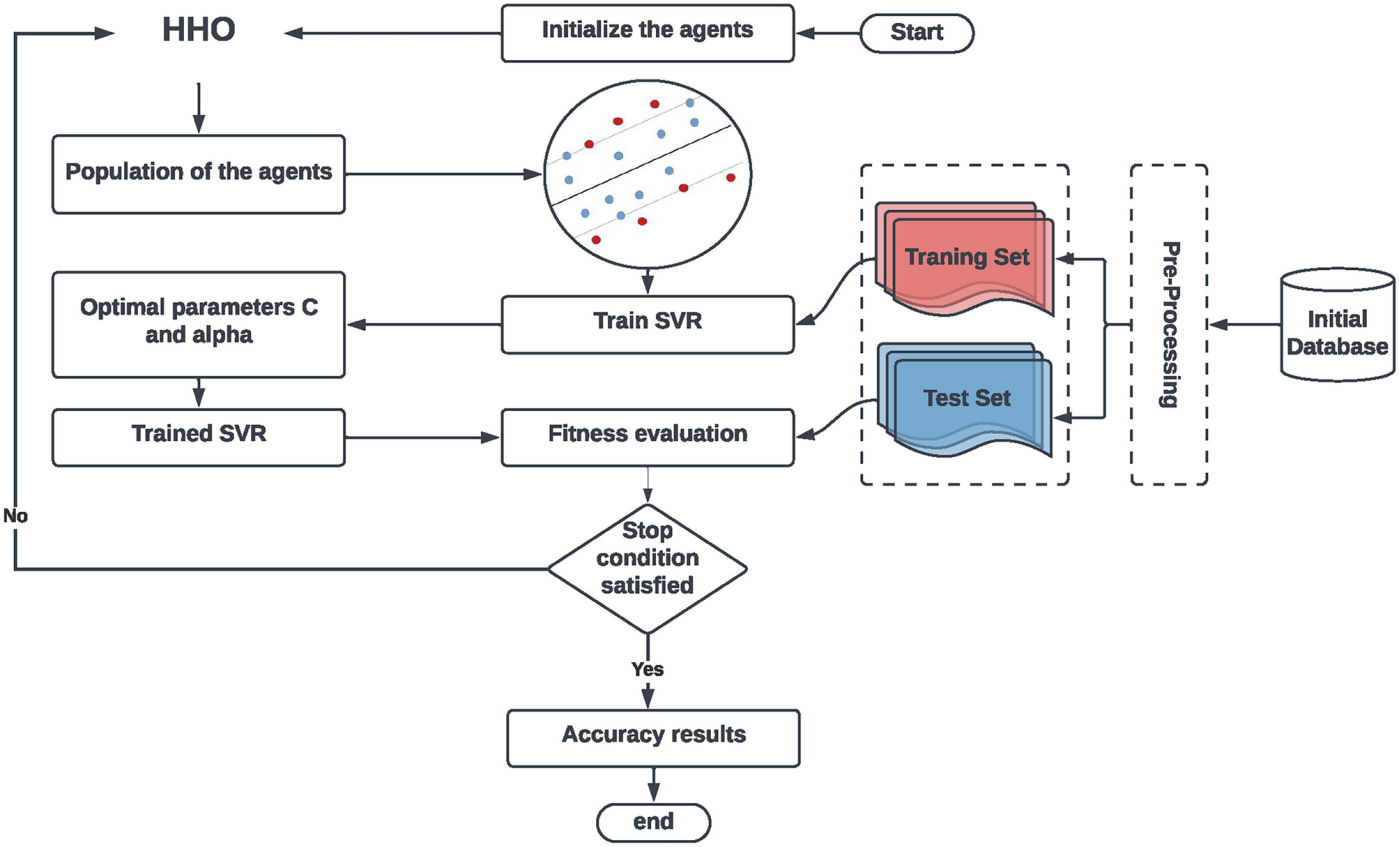
The Workflow of the suggested HHO-SVR model.

The HHO model combined with SVR for parameter tuning. Figure 6 represents The Workflow of the suggested HHO-SVR model, which illustrates the procedure into three main phases: (1) pre-processing, (2) parameter tuning, and (3) prediction and evaluation. Additionally, the pseudo-code for the HHO-SVR algorithm is provided in Algorithm 2.

**Algorithm 2:**
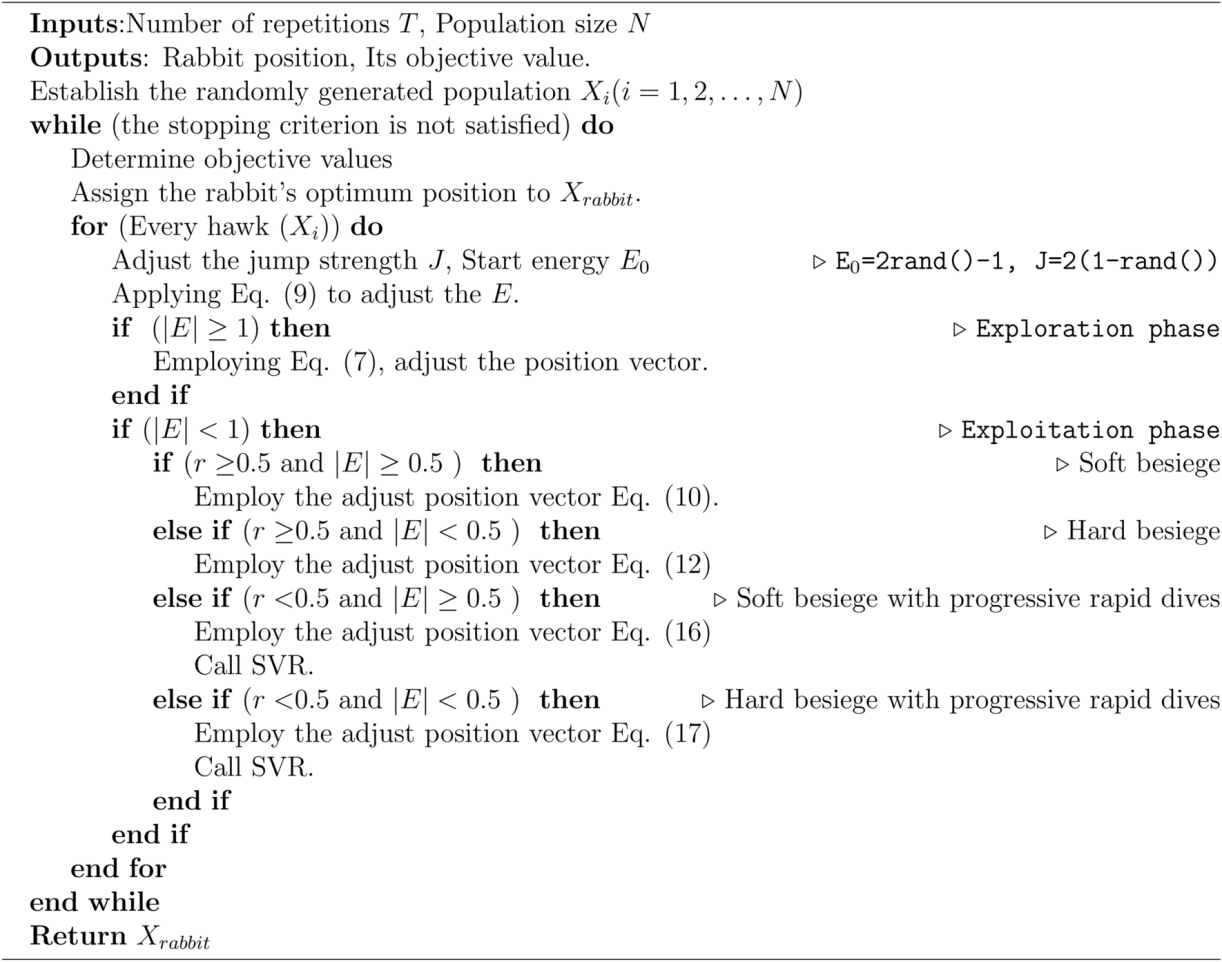
Pseudo-code of HHO-SVR.

### Objective function

To assess the efficacy of the proposed model, we subject the HHO solution to rigorous testing throughout the iterative process. The data is partitioned into training subset and testing subset during prediction. The MAPE serves as the objective function employed by HHO, acting as a statistical gauge of the precision of the prediction model. By excluding the minimum and maximum values and considering the average, MAPE provides a straightforward approach to addressing the problem. Its advantage lies in offering a clearer perspective on the actual prediction error, making it easily recognizable and quantifiable.

20 $$\begin{aligned} MAPE = \frac{1}{T}\sum \limits _{i = 1}^T {\left| {\frac{{{real_i} - {{fobj}_i}}}{{{real_i}}}} \right| } \end{aligned}$$

### Computational complexity of HHO-SVR

To evaluate the computational complexity of the proposed HHO-SVR hybrid model, we analyze the time and space requirements. For the HHO, the complexity of each iteration is *O*(*ND*), where *N* is the population size and *D* is the problem dimension. The SVR model involves solving a quadratic optimization problem with complexity $O(n^3)$, where *n* is the number of training samples. Thus, the overall complexity of HHO-SVR is:

21 $$\begin{aligned} O(T \cdot (ND + n^3)), \end{aligned}$$

where *T* is the number of iterations. This makes HHO-SVR computationally efficient for moderate data sizes.

### Theoretical justification for the proposed HHO-SVR

The improved performance of the HHO-SVR model can be attributed to the synergistic integration of HHO and SVR. The key features include:

- The *exploration and exploitation mechanisms* in HHO, such as soft and hard besiege strategies, enhance the search for optimal SVR parameters.
- The use of *Levy flight-based randomization* allows HHO to escape local minima, ensuring robust convergence.
- The *regularization and kernelization* capabilities of SVR improve model flexibility and generalization, leading to higher predictive accuracy.

The theoretical studies in^3^ and^71^ confirm that such hybridization can effectively mitigate overfitting and improve computational efficiency.

## Experimental results analysis and discussion

To mitigate potential bias in the selection of testing and training sets, this study employs 10-fold cross-validation for SVR. The proposed model’s efficiency is evaluated by comparing it with seven other well-known algorithms. All algorithms are implemented using Matlab and thoroughly documented studies to produce results. Specifically, the comparative algorithms are combined with a well-established machine learning model, SVR. Five $PM_{2.5}$ datasets are utilized to evaluate the proposed model’s effectiveness. Each comparative model is executed 10 times with 30 agents and 50 iterations.

The experiments are conducted on a machine with an Intel(R) Xeon(R) CPU E5-2698 v3 @ 2.30GHz (32 CPUs), 2.3GHz, 524288MB RAM, running Windows Server 2016 Datacenter, and MathWorks Matlab. The parameters for all evaluated approaches are defined as follows: 30 agents, a dimensionality of 2, 50 cycles, 10 independent trials, a minimum bound of 1, and a maximum bound of 1000. A selection of contemporary meta-heuristic algorithms, such as WOA, GWO, SSA, EO, HHO, HGSO, BMO, and MRFO, are considered for assessing the proposed technique. Each comparative algorithm employed an identical number of stochastic solutions. The objective function (*fobj*) is elaborated in earlier sections.

It is important to note that the parameter settings for all methods are summarized in Table 3. All tests were executed with an equivalent number of iterations (i.e., 50) and independent executions (i.e., 10).

**Table 3:** Parameter settings.

Algorithm	Parameter values	Termination Criteria
EO-SVR	$a_{1}=2, a_{2}=1$, and $GP=0.5$	Max iterations = 50
GWO-SVR	Wolves = 30,	Max iterations = 50
	*a* 0 : 2	
HGSO-SVR	Gases = 30, Clusters = 5,	Max iterations = 50
	$M_1= 0.1$, $M_2=0.2$,	
	$\beta = 1$, $\alpha =1$, *K* = 1,	
	$l_1=5E-03$, $l_2=100$, and $l_3=1E-02$	
HHO-SVR	Agents =30,	Max iterations = 50
	and $\beta = 1.5$	
MRFO-SVR	Agents =30	Max iterations = 50
BMO-SVR	Agents =30	Max iterations = 50
SSA-SVR	Agents =30	Max iterations = 50
WOA-SVR	Whales = 30,	Max iterations = 50
	*a* = 0 : 2,	
	and $a_2$ = $-2$ : $-1$	

### Data description

The dataset includes values for particulate matter levels ($PM_{2.5}$) generated by the Environmental Protection Agency’s (EPA) Downscaler model. These data are used by the Centers for Disease Control and Prevention’s (CDC) National Environmental Public Health Tracking Network to calculate air quality metrics. The dataset provides county-level information from 2001 to 2014, including maximum, median, mean, and population-weighted mean concentrations of $PM_{2.5}$. The Downscaler $PM_{2.5}$ dataset is derived from a Bayesian downscaling fusion model, combining $PM_{2.5}$ observations from the EPA’s Air Quality System with simulated data from the Models-3/Community Multiscale Air Quality (CMAQ) deterministic prediction model. Raw data processing involved extracting air quality monitoring data from the NAMS/SLAMS network, limited to Federal Reference Method (FRM) samplers. CMAQ data, from version 4.7.1 using the Carbon Bond Mechanism05 (CB-05), provides daily 24-hour average $PM_{2.5}$ concentrations on a 12 km x 12 km grid for the continental U.S. Additional processing standardized variable names and expanded FIPS variables into statefips and countyfips. Daily maximum, mean, median, and population-weighted values were computed for each county based on census tract estimates and 2010 U.S. Census tract-level population data. The Downscaler model synthesizes monitoring data and estimated $PM_{2.5}$ concentration surfaces from CMAQ to predict $PM_{2.5}$ levels across space and time, using optimal linear relationships to derive predictions and associated standard errors. Data can be found here (https://data.cdc.gov/Environmental-Health-Toxicology/Daily-County-Level-PM2-5-Concentrations-2001-2014/qjju-smys/about_data).

The data utilized in this experiment are summarized in Table 4, and the descriptive statistics are summarized in Table 5. Potential limitations of the used dataset include:

- *Geographic bias:* data do not represent all regions.
- *Temporal bias:* Seasonal variations may affect model performance across years.

Future studies should incorporate more diverse datasets to improve model generalizability.

**Table 4:** Description of dataset variables (24-hour average).

Variable	Description
STATEFIPS	State FIPS code
COUNTYFIPS	County FIPS code
YEAR	Year of prediction
DATE	Date (day-month-year) of prediction
$PM_{2.5}$-MAX-PRED	Maximum $PM_{2.5}$ concentration in $\mu g/m^3$
$PM_{2.5}$-MED-PRED	Median $PM_{2.5}$ concentration in $\mu g/m^3$
$PM_{2.5}$-MEAN-PRED	Mean $PM_{2.5}$ concentration in $\mu g/m^3$
$PM_{2.5}$-POP-PRED	Population-weighted $PM_{2.5}$ concentration in $\mu g/m^3$

**Table 5:** Descriptive Statistics on datasets.

Measure	1001	1003	1005	1007	1009
Mean	12.12437	11.15453931	11.4818384	12.28180649	13.42331558
Standard error	0.085314	0.077052276	0.083304949	0.094911277	0.10378677
Median	11.23851	10.22755	10.523719	11.180108	12.2115595
Mode	8.7698	12.1359	18.1112	10.1167	12.356719
Standard deviation	5.06668	4.601253288	4.973940345	5.665336729	6.152376078
Sample variance	25.67124	21.17153182	24.74008255	32.09604025	37.85173141
Kurtosis	3.653109	2.335702905	4.345759265	3.006152392	2.665884114
Skewness	1.376239	1.285206137	1.490456987	1.392239201	1.336003692
Range	42.11508	35.648372	48.424197	42.758139	42.303232
Minimum	2.6969	2.3414	2.2181	2.355192	2.3651
Maximum	44.81198	37.989772	50.642297	45.113331	44.668332
Sum	42762.64	39,777.08718	40,932.75391	43,760.07652	47,169.53096
Count	3527	3566	3565	3563	3514

### Data preprocessing

To improve forecasting results, we normalize the data using the minimal scale technique as described by^72^, following Eq. (22):

22 $$\begin{aligned} \hat{x_{i}} = \frac{x_{i}-\min (x)}{\max (x)-\min (x)}, \quad i\in [1,2,...,n] \end{aligned}$$

where $\hat{x_{i}}$ represents the normalized value within the *n* samples at index *i*.

### Evaluation metrics

The proposed approach is validated and assessed using the following metrics, based on the best objective value *fobj* achieved during run *i*:

- Average value of the objective function achieved by running the method *M* times. The average objective function is computed using the Eq. (23).
  23 $$\begin{aligned} Average = \frac{{\sum \nolimits _{i = 1}^M {fobj} }}{M} \end{aligned}$$
- Standard Deviation (*SD*) is used to measure the variance of the objective function calculated from running the method *M* times. It indicates the integrity and robustness of the model. Large *SD* values suggest inconsistent results, whereas smaller values indicate convergence of the algorithm to similar results across runs. *SD* is calculated using Eq. (24).
  24 $$\begin{aligned} SD = \sqrt{\frac{1}{{M - 1}}\sum \nolimits _{i = 1}^M {{{\left( {fobj - mean} \right) }^2}} } \end{aligned}$$
- The best objective function corresponds to the minimum objective value achieved by the method over *M* runs. This value is computed using Eq. (25).
  25 $$\begin{aligned} Best = \mathop {\min }\limits _{i = 1}^M \left( {fobj} \right) \end{aligned}$$
- The worst objective function is the highest objective value obtained from running the algorithm *M* times. This value is calculated using Eq. (26).
  26 $$\begin{aligned} Worst = \mathop {\max }\limits _{i = 1}^M \left( {fobj} \right) \end{aligned}$$
- CPU Time refers to the total time the central processing unit (CPU) utilizes to run the model *M* times.

### Results analysis and discussion

The empirical results of the HHO are compared with those of other hybrid approaches. Table 6 presents the results of the proposed model using Friedman’s ANOVA test in all data. The rank column indicates the algorithm’s ranking among the comparative methods based on Friedman’s ANOVA. The proposed approach demonstrated significant improvements, achieving the best results in three of the eight experiments.

**Table 6:** Results of the Friedman Test on the datasets and approaches.

Data	EO-SVR	GWO-SVR	HGSO-SVR	BMO-SVR	MRFO-SVR	SSA-SVR	WOA-SVR	HHO-SVR	P-value	Sigma
1001	7	1.6	5	2.9	4	6	8	**1**.**5**	2.704E−12	2.449E+00
1003	2	4.4	6.7	5.5	**1**	4.65	3.75	8	5.877E−11	2.448E+00
1005	2	3.5	6.1	6.9	4.8	3.7	8	**1**	3.743E−12	2.449E+00
1007	3.25	5	6.4	**1**.**4**	3.55	3.8	8	4.6	3.239E−08	2.439E+00
1009	3.8	5.4	7	**1**	3.5	5.3	8	2	7.279E−12	2.449E+00

The algorithms were ranked on the basis of their performance on several criteria: best, worst, average, standard deviation, and CPU time. The best performing algorithm is highlighted in boldface. Tables 7, 8, 9, 10, and 11 present the MAPE results, comparing the proposed approach with the seven recent approaches. The first column indicates the run number, while the subsequent columns represent the performed algorithms. Consequently, Tables 12, 13, 14, 15, and 16 exhibit the measured values for all methods, encompassing the best, worst, average, and standard deviation values, alongside CPU time, *C* value, and *Alpha* value.

**Fig. 7:**
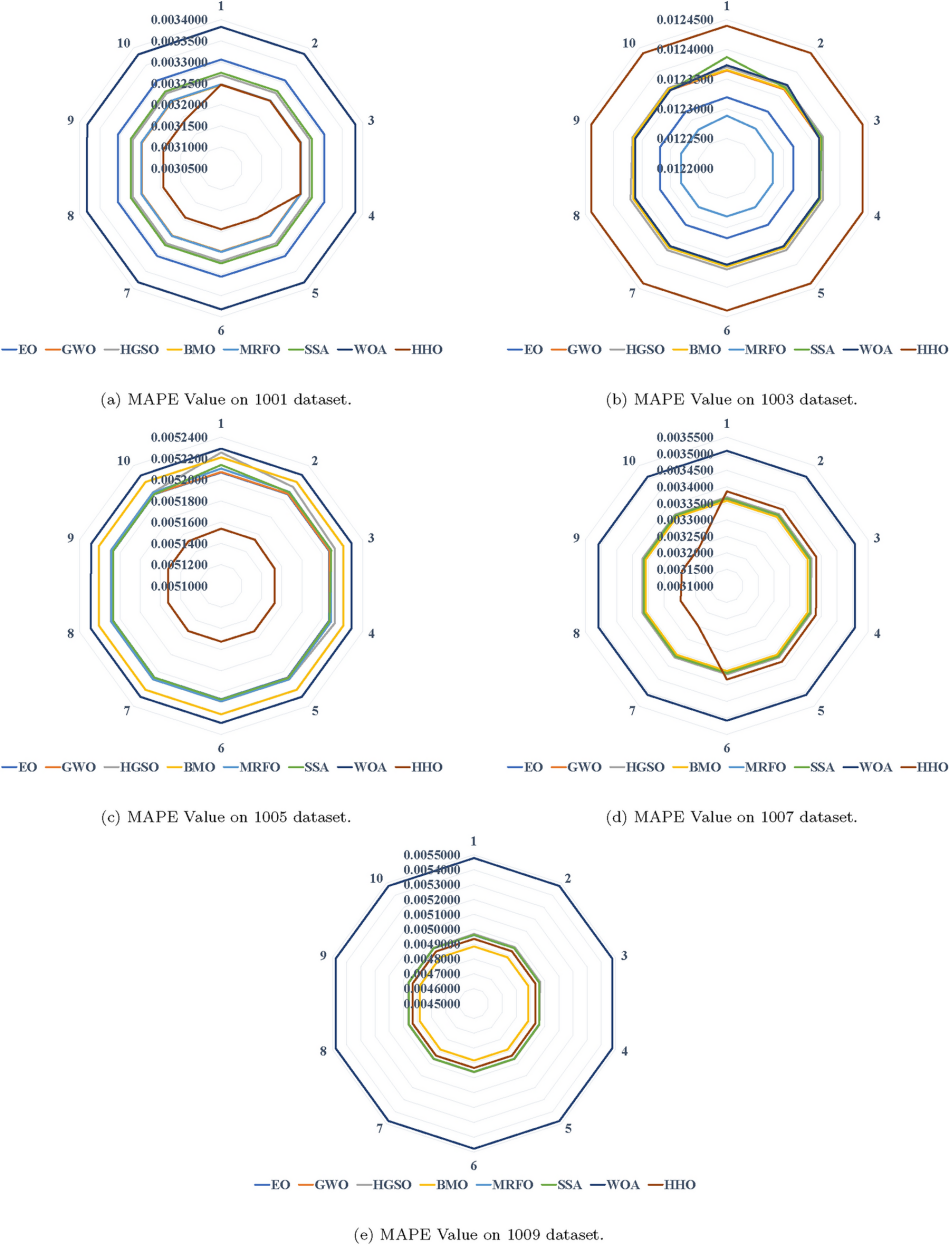
MAPE values on the proposed datasets.

In Table 9, it is evident that the hybrid SVR models achieved superior results with HHO compared to other comparative algorithms. The proposed approach displayed robust search capabilities, consistently nearing optimal solutions in various runs according to the MAPE metric. Similarly, Tables 7 and 10 underscore the consistent performance of the proposed hybrid approach in securing the best solution (Fig. 7).

Results varied across datasets. For instance, GWO demonstrated promising outcomes in the initial four runs, as depicted in Tables 7 and 8, whereas MRFO consistently outperformed other methods in all runs of dataset 1003. BMO closely followed HHO in achieving favorable results, as evident in Table 11, and exhibited superior performance in the initial six runs, as indicated in Table 10.

**Fig. 8:**
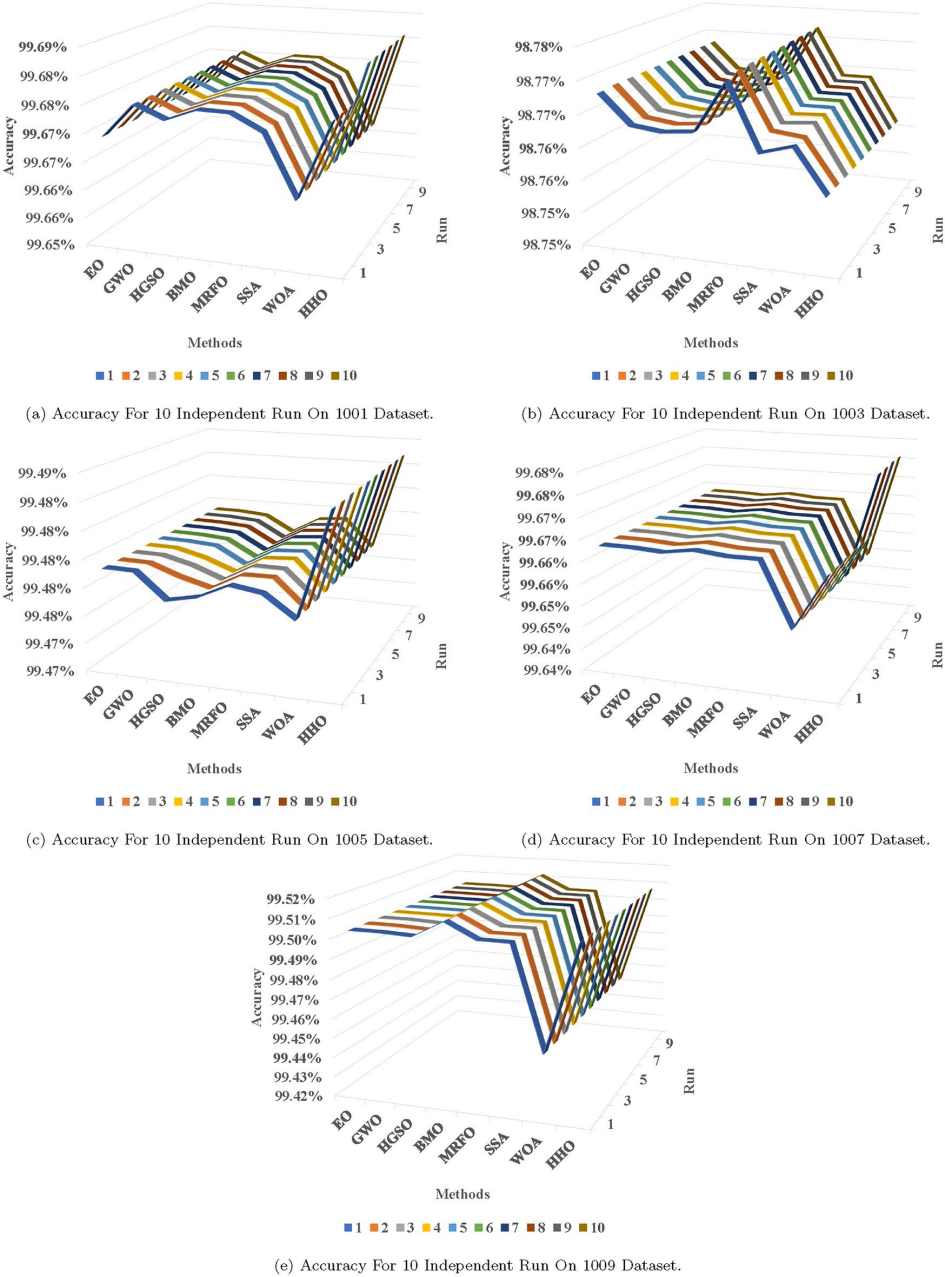
Accuracy on the proposed datasets.

Figure 8 illustrates the accuracy of different algorithms in datasets, showcasing the accuracy for each run. HHO consistently outperformed all other methods in Fig. 8c, as well as in some runs depicted in Fig. 8a and d.

In addition, radar charts representing MAPE across all time horizons among different models are depicted in Fig. 7. These charts summarize the error of all selected models for each dataset. HHO consistently demonstrated superior performance across almost all datasets, while WOA produced inferior results for most runs, as indicated by the radar lines surrounding the counterparts.

**Fig. 9:**
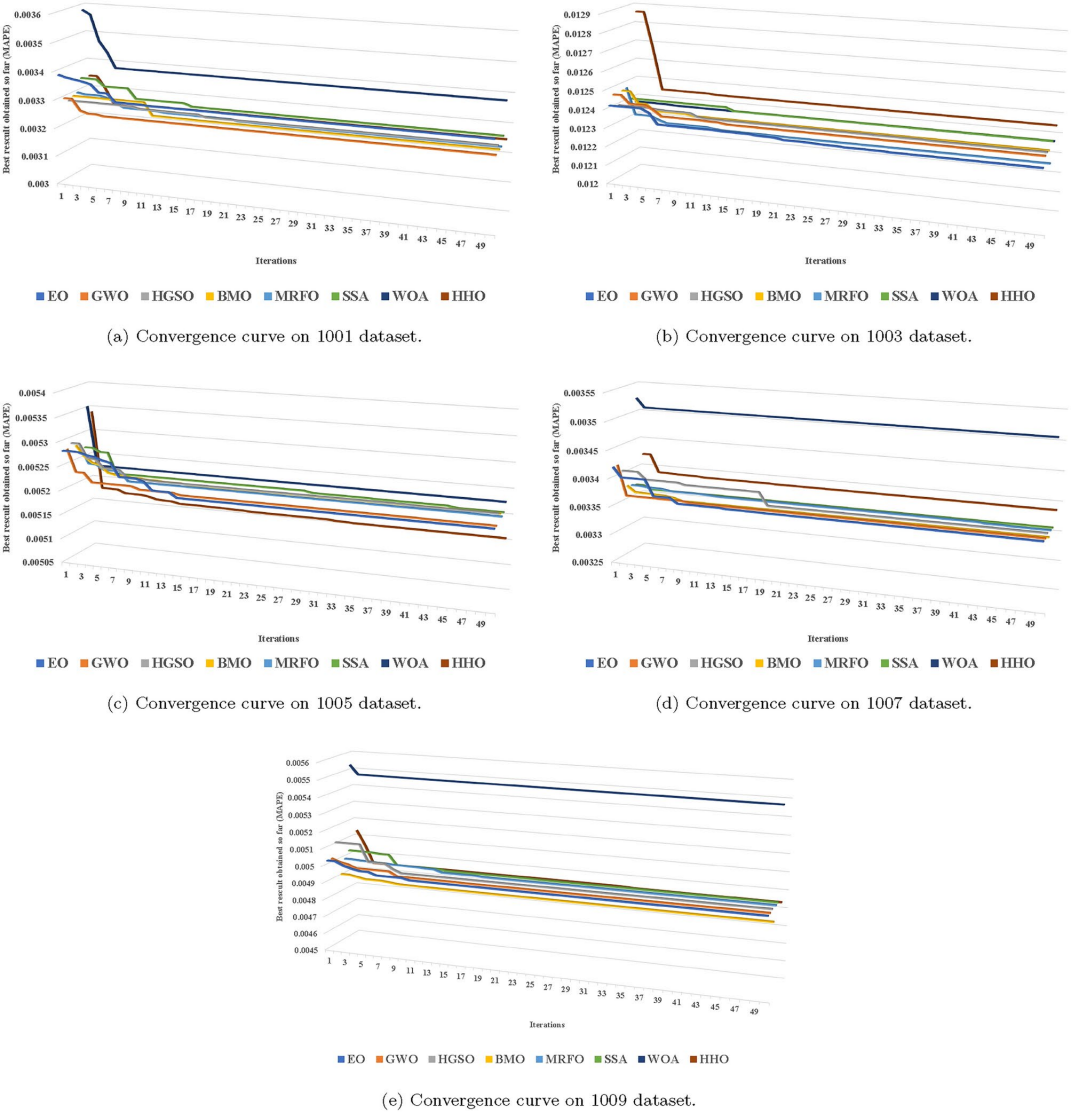
Sample of convergence curves on the proposed datasets.

Furthermore, Fig. 9 illustrates the convergence curves of various methods. As training iterations increase, the MAPE values calculated from different metaheuristics decrease. In Fig. 9a, WOA initially exhibited the highest MAPE results, while GWO outperformed other metaheuristic methods on the 1001 dataset in subsequent iterations. Similarly, in Fig. 9b, HHO initially showed the highest MAPE results, while EO outperformed other methods on dataset 1003 in subsequent iterations. Figure 9c–e also depict the convergence behaviour of different methods in the datasets 1005, 1007, and 1009, respectively.

**Table 7:** MAPE Comparative performance metric results between the proposed HHO-SVR model and other hybrid SVR approaches for dataset 1001 (50 Iterations and 10 Runs).

Run	EO-SVR	GWO-SVR	HGSO-SVR	BMO-SVR	MRFO-SVR	SSA-SVR	WOA-SVR	HHO-SVR
1	0.0033060	**0**.**0032463**	0.0032688	0.0032467	0.0032474	0.0032749	0.0033829	0.0032470
2	0.0033057	**0**.**0032462**	0.0032688	0.0032467	0.0032471	0.0032747	0.0033827	0.0032463
3	0.0033057	**0**.**0032458**	0.0032688	0.0032467	0.0032471	0.0032743	0.0033825	0.0032463
4	0.0033057	**0**.**0032458**	0.0032688	0.0032467	0.0032470	0.0032743	0.0033822	0.0032463
5	0.0033057	0.0032458	0.0032688	0.0032467	0.0032470	0.0032740	0.0033822	**0**.**0031945**
6	0.0033055	0.0032458	0.0032688	0.0032467	0.0032470	0.0032740	0.0033822	**0**.**0031937**
7	0.0033055	0.0032458	0.0032688	0.0032467	0.0032470	0.0032738	0.0033817	**0**.**0031934**
8	0.0033055	0.0032458	0.0032683	0.0032467	0.0032470	0.0032736	0.0033815	**0**.**0031934**
9	0.0033055	0.0032458	0.0032683	0.0032467	0.0032470	0.0032735	0.0033814	**0**.**0031930**
10	0.0033055	0.0032457	0.0032683	0.0032467	0.0032470	0.0032735	0.0033814	**0**.**0031921**

**Table 8:** MAPE Comparative performance metric results between the proposed HHO-SVR model and other hybrid SVR approaches for dataset 1003 (50 Iterations and 10 Runs).

Run	EO-SVR	GWO-SVR	HGSO-SVR	BMO-SVR	MRFO-SVR	SSA-SVR	WOA-SVR	HHO-SVR
1	0.0123195	0.0123645	0.0123702	0.0123655	**0**.**0122883**	0.0123871	0.0123733	0.0124397
2	0.0123176	0.0123639	0.0123702	0.0123655	**0**.**0122823**	0.0123675	0.0123729	0.0124396
3	0.0123176	0.0123639	0.0123702	0.0123655	**0**.**0122812**	0.0123675	0.0123633	0.0124396
4	0.0123176	0.0123637	0.0123702	0.0123655	**0**.**0122811**	0.0123648	0.0123627	0.0124396
5	0.0123176	0.0123637	0.0123702	0.0123655	**0**.**0122810**	0.0123625	0.0123622	0.0124396
6	0.0123176	0.0123635	0.0123702	0.0123655	**0**.**0122810**	0.0123625	0.0123622	0.0124393
7	0.0123176	0.0123632	0.0123702	0.0123655	**0**.**0122807**	0.0123624	0.0123620	0.0124393
8	0.0123176	0.0123630	0.0123702	0.0123655	**0**.**0122807**	0.0123621	0.0123620	0.0124393
9	0.0123176	0.0123630	0.0123669	0.0123655	**0**.**0122807**	0.0123621	0.0123620	0.0124393
10	0.0123176	0.0123625	0.0123669	0.0123655	**0**.**0122805**	0.0123620	0.0123620	0.0124393

**Table 9:** MAPE comparative performance metric results between the proposed HHO-SVR model and other hybrid SVR approaches for dataset 1005 (50 Iterations and 10 Runs).

Run	EO-SVR	GWO-SVR	HGSO-SVR	BMO-SVR	MRFO-SVR	SSA-SVR	WOA-SVR	HHO-SVR
1	0.0052068	0.0052073	0.0052256	0.0052210	0.0052105	0.0052139	0.0052293	**0**.**0051539**
2	0.0052068	0.0052073	0.0052152	0.0052210	0.0052093	0.0052090	0.0052292	**0**.**0051539**
3	0.0052068	0.0052073	0.0052127	0.0052210	0.0052088	0.0052090	0.0052292	**0**.**0051529**
4	0.0052068	0.0052073	0.0052127	0.0052210	0.0052088	0.0052074	0.0052292	**0**.**0051529**
5	0.0052068	0.0052073	0.0052090	0.0052210	0.0052088	0.0052074	0.0052292	**0**.**0051529**
6	0.0052068	0.0052073	0.0052090	0.0052210	0.0052088	0.0052071	0.0052292	**0**.**0051525**
7	0.0052068	0.0052073	0.0052090	0.0052210	0.0052088	0.0052069	0.0052292	**0**.**0051525**
8	0.0052068	0.0052073	0.0052090	0.0052210	0.0052088	0.0052069	0.0052292	**0**.**0051522**
9	0.0052068	0.0052073	0.0052090	0.0052210	0.0052088	0.0052069	0.0052285	**0**.**0051522**
10	0.0052068	0.0052071	0.0052090	0.0052210	0.0052088	0.0052069	0.0052285	**0**.**0051521**

**Table 10:** MAPE comparative performance metric results between the proposed HHO-SVR model and other hybrid SVR approaches for dataset 1007 (50 Iterations and 10 Runs).

Run	EO-SVR	GWO-SVR	HGSO-SVR	BMO-SVR	MRFO-SVR	SSA-SVR	WOA-SVR	HHO-SVR
1	0.0033643	0.0033647	0.0033689	**0**.**0033578**	0.0033652	0.0033650	0.0035089	0.0033862
2	0.0033643	0.0033646	0.0033689	**0**.**0033578**	0.0033643	0.0033650	0.0035084	0.0033859
3	0.0033643	0.0033646	0.0033689	**0**.**0033578**	0.0033643	0.0033650	0.0035082	0.0033843
4	0.0033643	0.0033646	0.0033679	**0**.**0033578**	0.0033643	0.0033644	0.0035080	0.0033837
5	0.0033642	0.0033646	0.0033679	**0**.**0033578**	0.0033643	0.0033644	0.0035080	0.0033837
6	0.0033642	0.0033646	0.0033679	**0**.**0033578**	0.0033641	0.0033641	0.0035080	0.0033836
7	0.0033642	0.0033644	0.0033679	0.0033578	0.0033641	0.0033641	0.0035080	**0**.**0032486**
8	0.0033642	0.0033643	0.0033679	0.0033578	0.0033641	0.0033640	0.0035080	**0**.**0032469**
9	0.0033640	0.0033642	0.0033679	0.0033578	0.0033641	0.0033640	0.0035080	**0**.**0032430**
10	0.0033640	0.0033642	0.0033666	0.0033578	0.0033641	0.0033640	0.0035080	**0**.**0032429**

**Table 11:** MAPE comparative performance metric results between the proposed HHO-SVR model and other hybrid SVR approaches for dataset 1009 (50 Iterations and 10 Runs).

Run	EO-SVR	GWO-SVR	HGSO-SVR	BMO-SVR	MRFO-SVR	SSA-SVR	WOA-SVR	HHO-SVR
1	0.0049599	0.0049606	0.0049677	**0**.**0048836**	0.0049609	0.0049618	0.0054783	0.0049347
2	0.0049599	0.0049605	0.0049677	**0.0048836**	0.0049606	0.0049604	0.0054772	0.0049338
3	0.0049599	0.0049605	0.0049677	**0**.**0048836**	0.0049598	0.0049603	0.0054772	0.0049338
4	0.0049599	0.0049605	0.0049623	** 0.0048836**	0.0049598	0.0049603	0.0054772	0.0049338
5	0.0049599	0.0049604	0.0049623	** 0.0048836**	0.0049598	0.0049603	0.0054772	0.0049338
6	0.0049599	0.0049603	0.0049623	**0**.**0048836**	0.0049598	0.0049603	0.0054772	0.0049338
7	0.0049599	0.0049603	0.0049623	**0**.**0048836**	0.0049598	0.0049603	0.0054772	0.0049338
8	0.0049599	0.0049600	0.0049623	**0**.**0048836**	0.0049598	0.0049602	0.0054772	0.0049338
9	0.0049599	0.0049600	0.0049623	**0**.**0048836**	0.0049598	0.0049602	0.0054772	0.0049338
10	0.0049599	0.0049600	0.0049623	** 0.0048836**	0.0049598	0.0049602	0.0054772	0.0049327

**Table 12:** MAPE statistics for 1001 dataset.

Methods	Best	Worst	Average	*SD*	CPU Time	C	Alpha	Rank
EO	0.0033055	0.0033889	0.0033066	0.0000074	69.4666568	1000	281	7
GWO	0.0032457	0.0032991	0.0032462	0.0000034	124.2312211	1000	650	2
HGSO	0.0032683	0.0032835	0.0032691	0.0000022	93.3155002	994	601	5
BMO	0.0032467	0.0032929	0.0032477	0.0000065	203.7472222	999	621	3
MRFO	0.0032470	0.0032985	0.0032478	0.0000049	95.6612414	993	608	4
SSA	0.0032735	0.0033440	0.0032751	0.0000069	96.1546419	999	423	6
WOA	0.0033814	0.0035842	0.0033832	0.0000132	26.1253467	998	100	8
**HHO**	**0**.**0031921**	** 0.0033400**	** 0.0032168**	**0**.**0000274**	**507**.**0908333**	**521**	**307**	**1**

**Table 13:** MAPE statistics for 1003 dataset.

Methods	Best	Worst	Average	*SD*	CPU Time	C	Alpha	Rank
EO	0.0123176	0.0124201	0.0123198	0.0000115	17.8029015	684	66	2
GWO	0.0123625	0.0124684	0.0123645	0.0000085	19.0358477	1000	47	5
HGSO	0.0123669	0.0124000	0.0123705	0.0000040	39.1187216	956	46	7
BMO	0.0123655	0.0124684	0.0123661	0.0000070	50.8555556	1000	52	6
**MRFO**	** 0.0122805**	**0**.**0124744**	** 0.0122831**	**0**.**0000100**	**24**.**2104032**	**975**	**64**	**1**
SSA	0.0123620	0.0124038	0.0123671	0.0000091	26.6693466	1000	47	4
WOA	0.0123620	0.0123771	0.0123653	0.0000049	24.9293342	1000	47	3
HHO	0.0124393	0.0128603	0.0124417	0.0000284	50.8356167	213	177	8

**Table 14:** MAPE statistics for 1005 dataset.

Methods	Best	Worst	Average	*SD*	CPU Time	C	Alpha	Rank
EO	0.0052068	0.0052823	0.0052080	0.0000086	47.3574331	1000	164	2
GWO	0.0052071	0.0052823	0.0052077	0.0000040	47.1367420	1000	163	4
HGSO	0.0052090	0.0052907	0.0052135	0.0000083	66.6136964	972	167	6
BMO	0.0052210	0.0052823	0.0052214	0.0000037	74.2916667	888	190	7
MRFO	0.0052088	0.0052754	0.0052094	0.0000040	35.3579964	973	136	5
SSA	0.0052069	0.0052709	0.0052093	0.0000063	38.9735247	1000	166	3
WOA	0.0052285	0.0053537	0.0052293	0.0000056	35.9075620	1000	100	8
**HHO**	**0**.**0051521**	**0**.**0053394**	**0**.**0051537**	**0**.**0000087**	**315**.**4734056**	**335**	**226**	**1**

**Table 15:** MAPE statistics for 1007 dataset.

Methods	Best	Worst	Average	*SD*	CPU Time	C	Alpha	Rank
EO	0.0033640	0.0034207	0.0033648	0.0000044	113.8061068	1000	574	4
GWO	0.0033642	0.0034207	0.0033647	0.0000025	112.6148437	1000	572	6
HGSO	0.0033666	0.0034069	0.0033692	0.0000051	90.0221714	978	583	7
BMO	0.0033578	0.0033764	0.0033579	0.0000011	186.5833333	787	576	2
MRFO	0.0033641	0.0033739	0.0033646	0.0000013	103.7009238	999	573	5
SSA	0.0033640	0.0033713	0.0033645	0.0000006	115.8633256	999	575	3
WOA	0.0035080	0.0035252	0.0035082	0.0000008	48.2469431	1000	100	8
**HHO**	**0**.**0032429**	**0**.**0034207**	**0**.**0033308**	**0**.**0000678**	**472**.**2213889**	**934**	**429**	**1**

**Table 16:** MAPE statistics for 1009 dataset.

Methods	Best	Worst	Average	*SD*	Time (H)	C	Alpha	Rank
EO	0.0049599	0.0050321	0.0049606	0.0000057	115.8019583	1000	570	4
GWO	0.0049600	0.0050321	0.0049610	0.0000052	116.2327811	1000	519	5
HGSO	0.0049623	0.0051158	0.0049654	0.0000144	114.3111473	990	521	7
**BMO**	**0**.**0048836**	**0**.**0049141**	**0**.**0048839**	**0**.**0000024**	**275**.**2083333**	**1000**	**717**	**1**
MRFO	0.0049598	0.0049935	0.0049607	0.0000042	109.5209263	999	570	3
SSA	0.0049602	0.0050321	0.0049614	0.0000077	117.8218834	1000	567	6
WOA	0.0054772	0.0055324	0.0054775	0.0000025	38.8181049	1000	100	8
HHO	0.0049327	0.0051315	0.0049348	0.0000103	288.5619450	862	798	2

The HHO-SVR model exhibits enhanced performance relative to alternative optimization algorithms, attributed to its distinctive integration of HHO and SVR methodologies. The HHO algorithm efficiently balances exploration and exploitation in parameter tuning by employing mechanisms like soft and hard besiege strategies, along with Levy flight-based randomization. The mechanisms facilitate HHO’s exploration of various areas within the search space while concentrating the search on promising solutions, thereby ensuring effective optimization of SVR hyperparameters. The adaptability of HHO-SVR facilitates enhanced prediction accuracy, especially in high-dimensional parameter spaces where conventional optimization algorithms frequently encounter stagnation. HHO effectively addresses the complexities of the PM2.5 forecasting problem through dynamic adjustments of escape energy and jump strength.

The model’s performance undergoes further validation via statistical tests and supplementary metrics. Friedman’s ANOVA test indicates that HHO-SVR consistently achieves the highest predictive accuracy across various datasets, with statistically significant p-values (<0.01) noted in the majority of instances. The comparative analysis of convergence curves indicates that HHO-SVR demonstrates a faster convergence rate than alternative algorithms, reaching optimal solutions in fewer iterations. Furthermore, HHO-SVR exhibits enhanced robustness, characterized by diminished variability in MAPE across various iterations, signifying a reduction in overfitting and consistent performance. The findings, along with HHO-SVR’s superior performance in MAPE and CPU time metrics compared to competing algorithms, highlight its efficacy in air quality forecasting and its potential for wider environmental applications.

## Conclusion and future directions

In this study, we introduced an innovative hybrid approach for predicting $PM_{2.5}$ concentrations by combining Support Vector Regression (SVR) with the Harris Hawks Optimization (HHO) algorithm called the HHO-SVR model. Through experimentation and comparison with seven other optimization algorithms, the proposed HHO-SVR demonstrated promising performance in specific scenarios. Furthermore, the proposed HHO-SVR model consistently showed superior predictive accuracy, achieving the best results in three of the eight experiments in five distinct $PM_{2.5}$ datasets. Statistical analysis using Friedman’s ANOVA test affirmed the robustness and high ranking of the HHO-SVR model, highlighting its effectiveness in diverse environmental contexts. However, the hybrid HHO-SVR model consistently outperformed competing approaches, demonstrating its potential for practical application in environmental monitoring and management. In conclusion, this study presented a promising avenue for improving prediction precision $PM_{2.5}$ by using the synergistic benefits of SVR and HHO. The demonstrated superiority of the proposed HHO-SVR model underscores its potential to advance environmental forecasting capabilities, enabling informed decision making for sustainable environmental management. In future work, the proposed HHO-SVR model will be applied to solve another climate change issue, such as forecasting the increase in temperatures and other factors of climate change.

## Data Availability

The data sets provided during the current study are available: https://data.cdc.gov/Environmental-Health-Toxicology/Daily-County-Level-PM2-5-Concentrations-2001-2014/qjju-smys/about_data.
